# Effect of 8 weeks badminton session on cardiovascular and neuromuscular functions among older adults in United Arab Emirates: a quasi-experimental study

**DOI:** 10.12688/f1000research.142339.1

**Published:** 2023-11-28

**Authors:** Animesh Hazari, Sondos Jalgoum, Praveen Kumar Kandakurti

**Affiliations:** 1 College of Health Sciences, Gulf Medical University, Ajman, United Arab Emirates; 2 Physiotherapy, College of Health Sciences, Gulf Medical University, Ajman, United Arab Emirates; 3 College of Health Sciences, Gulf Medical University, Ajman, United Arab Emirates

**Keywords:** Badminton, Cardiovascular, Neuromuscular, older adults, Communicable disease, UAE

## Abstract

**Background:**

Older adults (40-70 years) are the most susceptible age group for developing cardiovascular, and neuromuscular disorders due to a lack of physical activities. The engagement of older adults in physical activities such as badminton can improve their neuromuscular function. Thus, this study aimed to analyze the effects of badminton on cardiovascular & neuromuscular function among older adults with and without non-communicable diseases in the United Arab Emirates.

**Methods:**

A total of 120 participants were recruited and divided into three groups: Two interventional groups which consisted of participants with non-communicable disease (WCN, N=40), and participants without the non-communicable disease (WICN, n=40), and one non-interventional group (NIC) as healthy control participants. Groups with and without non-communicable diseases engaged in badminton (45-60 minutes per session, thrice a week for two months) as per the specific inclusion and exclusion criteria.

**Results:**

The findings of the study indicated that there was a significant improvement in cardiovascular and many neuromuscular variables within and between the groups (p≤0.05) with maximum changes in participants with non-communicable diseases.

**Conclusions:**

Engagement in sports like badminton can help to overcome the non-communicable disease burden. The immediate impact can be seen with the introduction of such interventional sports activities on a larger scale. Since the improvement was seen to be much better in the participants with non- communicable diseases, it could help to reduce the burden of non-communicable diseases.

**Clinical Trial Registry, India registration:**

REF/2022/02/051455 (08/02/2022)

## Introduction

The true meaning of fitness for humankind has a wider spectrum. It can be correlated with being free from various disorders including cardiovascular, musculoskeletal, neurological, and psychosocial conditions, and thus lead to an enhanced quality of life which can be significantly achieved by incorporating adequate physical and sports activities ( Marquez *et al*. 2020). Sports and physical activities go hand in hand and thus not only can prevent the progression of non-communicable diseases but could help in reversing the course of diseases as well ( Saqib *et al*. 2020, Hazari and Kumar 2023). Research from a variety of scientific fields suggests that physical activity in nature and feelings of connection to nature enhance psychological health and well-being ( Lawton *et al*. 2017). Outdoor sports activity has benefits in lowering the levels of stress, anxiety, depression, and parameters related to non-communicable diseases ( Saqib *et al*. 2020). It is well known that the presence of non-communicable disease has a higher correlation with morbidity related to cardiovascular disorders ( Hadlaq *et al*. 2022), and aerobic training directly reflects the cardiovascular as well as neuromuscular fitness of an individual. Given that adherence to physical activities is poor among older adults in form of general aerobic training ( Rivera *et al*. 2019), engaging them in outdoor sports activities could be more useful to dampen the course of the non-communicable disorders, thereby preventing future complications. In the present study, we have operationally defined older adults within the age group of 40 to 70 years as there is inconsistency in clear sub-classification for adults ( Petry 2002). In addition to aging effects, loss of muscle strength, muscle tightness, reduced agility, poor balance, hand-eye coordination, etc. are very commonly seen as a factor of reduced physical activity among older adults ( Hunter *et al*. 2016, Wilkinson *et al*. 2018). Literature also suggests that the ill effects of aging can be controlled with physical activity promotion ( Bernardelli *et al*. 2019).

Sports play a significant role in the promotion of physical activity. Badminton holds a significant position in popularity for outdoor sports globally ( Sondos *et al*. 2023). Badminton targets the cardiovascular, musculoskeletal, and neurological systems significantly. The game requires the constant engagement of the individual which also improves their psychological and mental abilities and makes them more social. Badminton is also widely accepted by various age groups allowing the use of it as a priority sport to enhance physical activity. In addition to the non-contact nature which made it safer and suitable during the Covid-19 pandemic. The non-contact nature of the sport also makes it suitable for encouraging higher female participation in the United Arab Emirates.

Research has suggested that Badminton puts significant demands on the cardiovascular and neuromuscular systems ( Cabello *et al*. 2022). The aerobic nature of the sport can specifically target cardiovascular endurance and help in weight management for obesity. The most common neuromuscular parameters which can be targeted through Badminton include agility, speed, power, muscle strength, muscle length, muscle recruitment pattern, and peak force ( Cinthuja *et al*. 2015). In addition, it also enhances neural functions such as body balance, coordination, response time, reaction time (hand-eye-coordination), and proprioceptive feedback ( Hülsdünker *et al*. 2019). The combination of such improvements ultimately has a positive impact on well-being overall. Thus, the purpose of the study was to analyze the effects of Badminton sports on the cardiovascular and neuromuscular systems among older adults of the United Arab Emirates (UAE). The proposed research was focused on improving the physical activity and well-being of older adults in the UAE and thereby reducing the burden of non-communicable diseases in the country. In UAE, the prevalence of non-communicable diseases such as diabetes, hypertension, obesity, and anxiety disorders is very high, and physical inactivity is the major risk factor ( Mamdouh *et al.* 2023). The engagement of older adults in Badminton sports could prevent the risk of non-communicable diseases. In addition, for people already suffering from such disorders, the course of the disease could be ceased or reversed. The objectives of the study were as follows:

1. To analyze the changes in cardiovascular and neuromuscular fitness parameters for older adults with non-communicable diseases in UAE.
2. To compare the pre- and post-cardiovascular and neuromuscular changes following 8 weeks badminton session for older adults with non-communicable disease against age and gender matched participants without non- communicable disease.
3. To analyze the effects of 8-week Badminton session on Cardiovascular and Neuromuscular functions for older adults (with and without non-communicable disease) to age matched to healthy control.

## Methods

### Study design and setting

**Figure 1.**
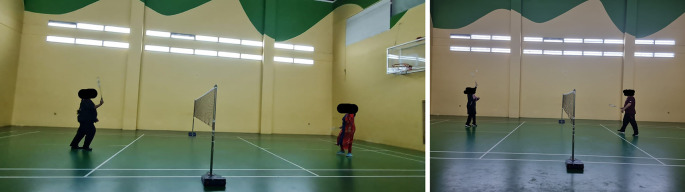
Participants using Indoor Badminton court at Thumbay University, UAE. Written informed consent was obtained from the participants for the use and publication of this image.

A Quasi experimental study was conducted from March 2022 till October 2023 at the Indoor Badminton Court ( Figure 1).

### Ethical considerations

The study was approved by the Institutional Ethics Committee, Thumbay Hospital Gulf Medical University (Ref no- IRB/COHS/FAC/67-sep 2021). Written informed consent was obtained from all participants.

### Participants

A total of 120 participants were divided equally into three groups. Two interventional groups which consisted of participants with non-communicable disease (WCN, n=40), and participants without the non-communicable disease (WICN, n=40), and one non-interventional group (NIC) as healthy control participants were recruited in the study under the purposive sampling method. The participants for the non-communicable group were approached through medical records and contacts at the Thumbay Hospital, Gulf Medical University. The participants in the health and without non-communicable disease were approached via flyers, and personal local contacts. The inclusion and exclusion criteria were as follows:

Inclusion criteria: Age group- 40 to 70 years, both male and female (self-reported gender analysis), participants with non- communicable disease (diabetes mellitus, hypertension, and obesity only), age and gender- matched participants without the non-communicable disease and healthy control. The NIC group were selected for baseline comparison as a subgroup of participants without non-communicable diseases who agreed to participate. Simple randomization was using chit method was employed to categorize participants as WICN and NIC.

Exclusion criteria: Any joint disorders, cancer, neurologically unstable or prior physically active individuals, participants on regular consumption of alcohol or any medication which could affect their abilities and performance during the badminton session.

### Procedure

A total of 249 participants were screened rigorously before we reached the desired sample size of 40 in each group, making a total of 120 participants based on the inclusion and exclusion criteria. The sample size calculation was done by the statistician using the formula for comparison of means (joint range of motion at the shoulder). The total calculated sample size was 98, however 120 samples were recruited considering a dropout rate of 20%. The categorization for the presence or absence of non-communicable disease was done based on the participant’s past medical history and clinical investigatory findings such as blood sugar level, blood pressure levels, and lipid profile.

The pre-participation screening questionnaire (PAR-Q) was administered to all participants to minimize the exercise-related risk and rule out any prior cardiovascular disorders before engaging in the badminton session. The validity of PARQ among the elderly population has been given in a study by de Oliveira Luz *et al.* (2007, URL - (16) (PDF) Validity of the physical activity readiness questionnaire (PAR-Q) in elder subjects (researchgate.net)). For participants with communicable diseases such as diabetes and hypertension appropriate precautionary measures were taken to prevent and manage exercise- related complications. The experimental group participants (WCN and WICN) were required to engage in the supervised Badminton game for 45-60 minutes per session, thrice a week for two months at mild to moderate exercise intensity, monitored on Rate of Perceived Exertion Scale (RPE 0-10). Participants in the NIC group were allowed to continue their daily routine activities as their regular choice and comfort. The age, gender, and level of participants were matched by the supervising physical therapist.

The medical emergency and requisite arrangements were available on the University Hospital premises on all days during the session. In addition, a badminton coach was also engaged in the session for any technical help in the sport.

### Outcome measures

The primary outcome variables and measures were focused on the cardiovascular, and neuromuscular components as listed below:

Cardiovascular Parameters:

‐ 6-minute walk test: 6-minute walk distance (6MWD) and estimated VO _2_ peak
‐ Rate of Perceived Exertion (RPE) on 0-10 scale

Neuromuscular Parameters:

‐ Agility using Modified T-test
‐ Lower Limb Joint Power using Force plates and motion analysis system (BTS GAITLAB, BTS Bioengineering Corp., U.S.A)
‐ Muscle peak force for Quadriceps and Hamstring using Isokinetic device (ISOMOVE, TecnoBody, Italy)
‐ Proprioception using ProKin 252, Tecnobody, Italy
‐ Balance (Single leg stance) using ProKin 252, Tecnobody, Italy
‐ Reaction time using D wall, Tecnobody, Italy
‐ Hand eye-coordination using D wall, Tecnobody, Italy
‐ Muscle strength using Manual Muscle Testing (Oxford Grading)
‐ Muscle length for lower limb using physical therapy clinical examination
‐ Joint Range of Motion for Shoulder and Lower Limb using physical therapy clinical examination

### Data acquisition

**Figure 2.**
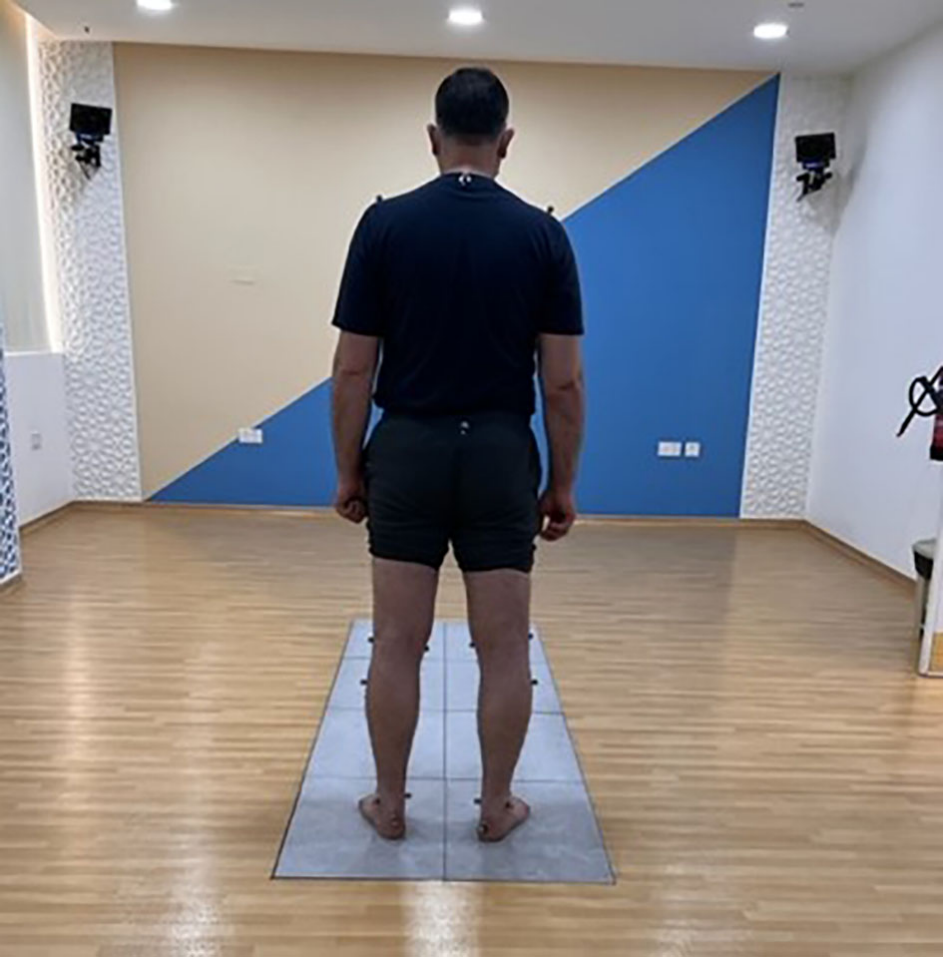
Showing Halen Hayes MM model for Gait Analysis, TPTR, GMU. Written informed consent was obtained from the participants for the use and publication of this image.

The lower limb joint power was calculated using the inverse dynamics laws ( Muro *et al*. 2014) at the BTS force plate during the gait analysis with 8 video cameras (BTS SMART-DX system). Retroreflective markers were placed on the joint centers using the Halen-Hayes MM model ( Figure 2). All the participants were asked to walk at their normal speed from a set start line. Three successful trials were taken and the peak power at the hip, knee, and ankle were taken for data analysis.

**Figure 3.**
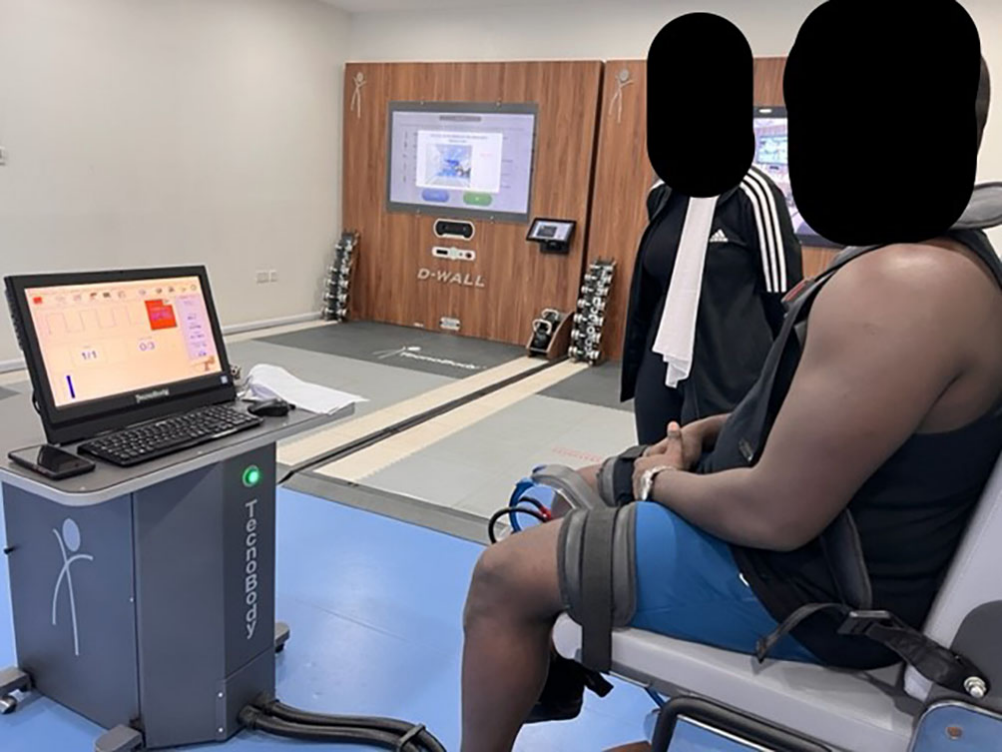
Showing peak muscle force analysis at Isomove Isokinetic Device, TPTR, GMU. Written informed consent was obtained from the participants for the use and publication of this image.

The peak muscle force for Quadriceps and Hamstring both were calculated through the isokinetic device ( Risberg *et al*. 2018) with a flexion angle set at 90 degrees and extension at 0 degrees (Torque 60 deg. Sec-1) as shown in Figure 3. Participants were instructed to generate their max force of push for the Quadriceps and pull for the Hamstring. Three complete cycles were taken, and the peak muscle force was used for data analysis.

**Figure 4.**
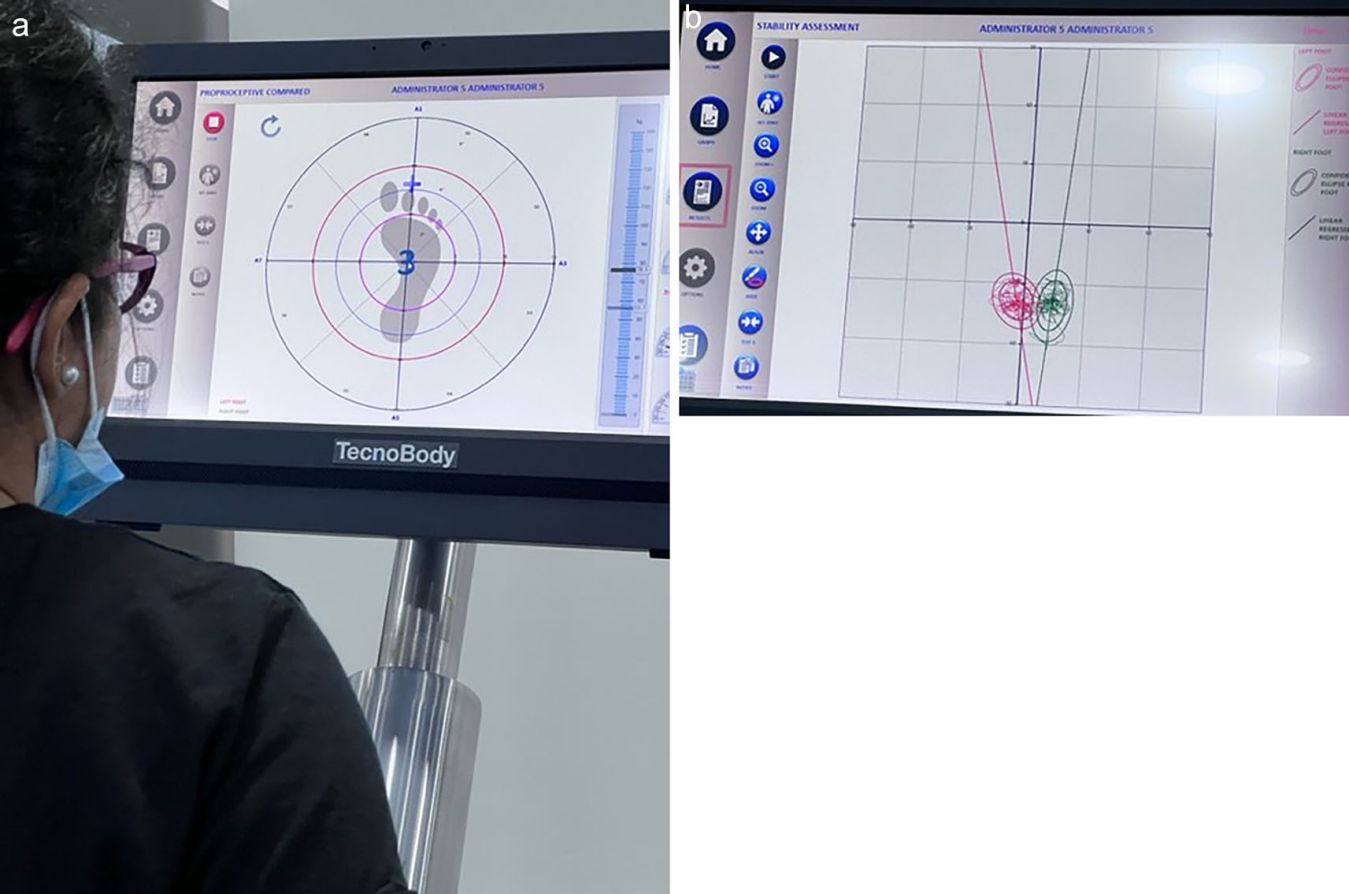
a. Proprioception analysis, b. balance analysis. Written informed consent was obtained from the participants for the use and publication of this image.

The proprioception and balance were calculated using the ProKin device ( Amico *et al*. 2014) while standing on the embedded vowel board as shown in Figure 4a and 4b. The participants were asked to stand barefoot with eyes closed and with eyes open for stability assessment on both the right and left lower limbs respectively. The stability was assessed in the Anteroposterior (A-P) and Medio- Lateral (M-L) directions. Proprioception was also assessed for individual limbs while creating a 360-degree arc using the different compartments of the foot stable over the platform.

**Figure 5.**
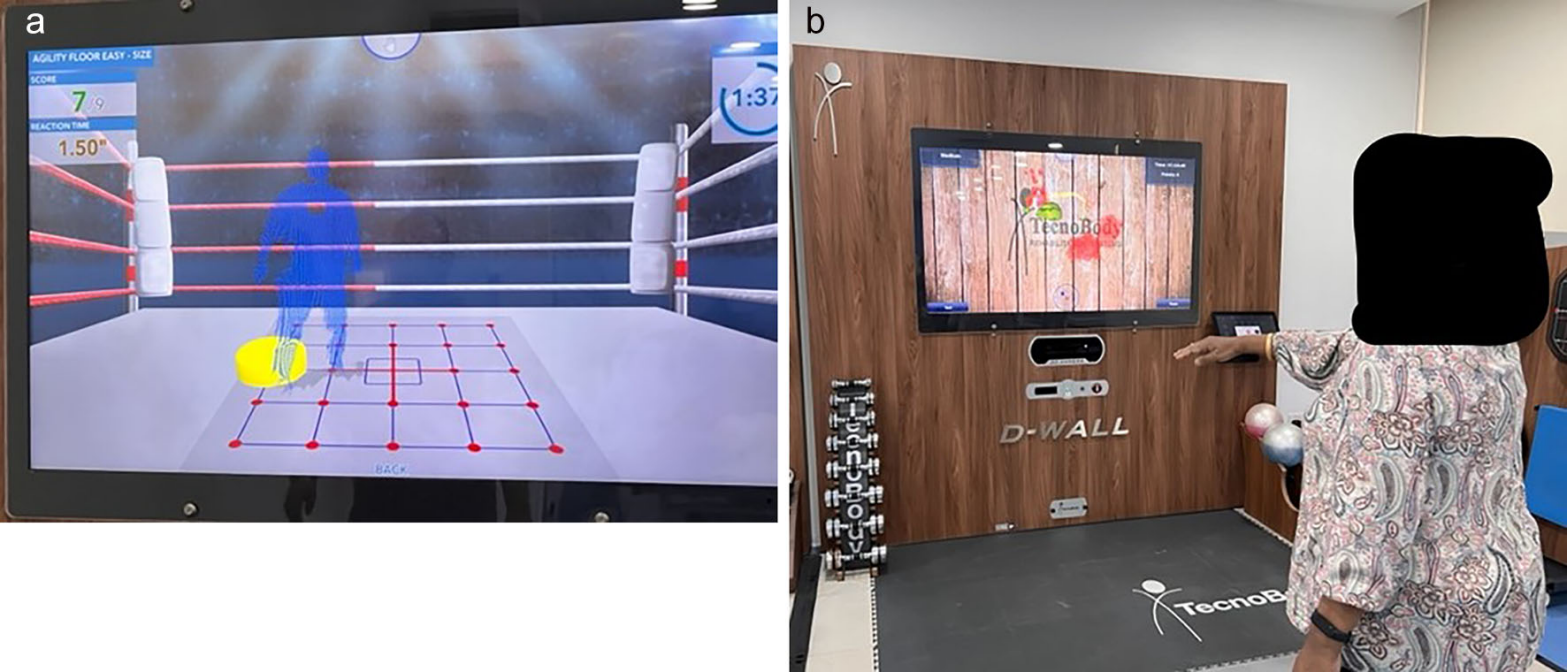
a. Reaction time assessment, b. hand eye coordination assessment. Written informed consent was obtained from the participants for the use and publication of this image.

The reaction time and hand-eye coordination were calculated using the D Wall system ( Hazari *et al*. 2021) where specific tasks were given to all participants ( Figure 5a and 5b). For instance, the reaction time was checked with the number of correct shifts in all the quadrants of the floor whereas the hand- eye coordination was assessed using the number of fruits cut. For the task, the time limit was two minutes.

The assessment for the six-minute walk test using the standard guidelines given by the American Thoracic Society over a 15 m walkway ( Enright 2003), Modified T-Test ( Vallejos *et al*. 2020), muscle length, and muscle strength was done as per the given physical therapy clinical settings.

### Data analysis

SPSS 21 was used for data analysis for descriptive and analytical tests. Since the data were normally distributed, we conducted analysis of Variance (ANOVA). *Post-hoc* analysis was done to establish the significant marginal difference among the groups. The effect size was calculated for post-session changes between the group with Cohen’s d.

Dropouts:

In total there were six dropouts (2 from the WCN, and 4 from WICN group). Four participants left the study during the second week and two participants could not continue after 6 weeks and thus were excluded from data analysis.

## Results and discussion

**Figure 6.**
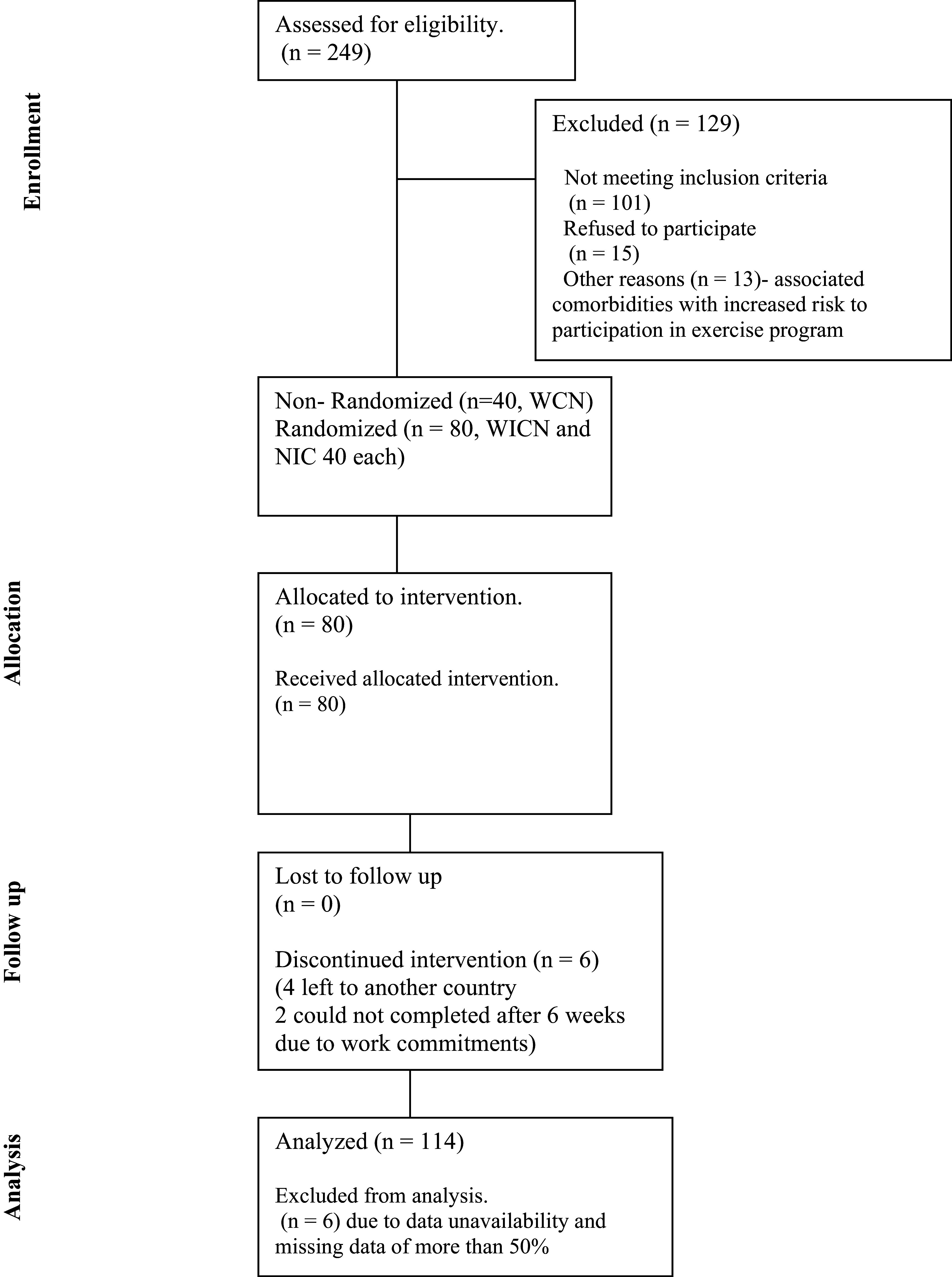
Flow diagram for participants enrollment, allocation, and analysis. Written informed consent was obtained from the participants for the use and publication of this image.

The results of participants recruitment, allocation to intervention and drop-out have been presented in the flow diagram ( Figure 6). The important findings of the study have been represented below using tables for within and between groups comparisons. It was evident that Badminton session improved cardiovascular and neuromuscular functions for older adults with and without non-communicable disease both (p <0.05) ( Hazari, 2023). The effect size was significantly higher for the group with non-communicable disease.

In the present study, 114 participants were taken for data analysis (out of 120) since six participants dropped out due to inability to continue the badminton session for personal reasons. The demographic data for each group has been presented in Table 1a, Table 1b, and Table 1c, respectively, and the mean difference in age and body mass index (BMI) was statistically insignificant (p=0.12 for WCN and WICN, p=0.38 for WICN and NIC, p=0.21, for WCN and NIC) suggesting that groups were comparable with similar physical characteristics. Table 2 represents a within-group analysis of neuromuscular parameters for participants with non-communicable diseases (WCN). The mean range of motion (pre-session) for shoulder flexion was 178 degrees which changed to 180 degrees post-badminton session. Similar findings were observed for other shoulder motions such as extension, internal and external rotation. However, a statistically significant difference was found only for flexion and external rotation range of motion (p=0.05 and 0.002, respectively). It is well known that non-communicable diseases such as diabetes lead to shoulder stiffness with a capsular pattern. Previous studies have reported a mean reduction of 10 to 17 degrees for shoulder flexion, abduction, and external rotation among patients with diabetes ( Cole *et al*. 2009). In our study, there was no reported case of frozen shoulder but the lower baseline values for mean shoulder range of motion could be suggestive of shoulder stiffness in its earlier phases due to the presence of non-communicable disease. The improvement in shoulder range of motion could be attributed to the stretching of muscle and soft tissues within the joint including the capsule over the period while engaging in badminton sessions. The range of motion would have improved with the shoulder diagonal pattern of movements as per the Proprioceptive Neurofacilitation Techniques (PNF) used in racket sports such as badminton.

**Table 1a.** Baseline demographic characteristics of participants with non-communicable diseases (WCN).

Participants (n=38) DM=32 Obesity=5 Anxiety=1		Age		BMI	
Male	Female	Mean	S.D.	Mean	S.D.
27	11	57.43	4.19	23.29	2.13

**Table 1b.** Baseline demographic characteristics of participants without non-communicable diseases (WICN).

Participants (n=36)		Age		BMI	
Male	Female	Mean	S.D.	Mean	S.D.
30	6	56.71	4.42	22.81	2.06

**Table 1c.** Baseline demographic characteristics of non- interventional healthy control participants (NIC).

Participants (n=40)		Age		BMI	
Male	Female	Mean	S.D.	Mean	S.D.
32	8	57.02	3.39	22.27	3.11

**Table 2.** Neuromuscular finding for participants with non-communicable disease (WCN).

Variables			Pre-Sessions		Post-Sessions		P value Sig (≤0.05)
			Mean	S.D.	Mean	S.D.	
**Shoulder Range of Motion (Degree)**	Flexion		178	3.68	180	0.91	**0.05**
	Extension		58.33	3.08	60	0.54	0.06
	Abduction		178	4.17	179	1.29	0.08
	External Rotation (ER)		84.67	4.81	89.67	1.29	**0.002**
	Internal Rotation (IR)		73.67	7.89	87.67	6.78	0.07
**Muscle Peak Force (Nm)**	Quadriceps		66.18	22.16	73.21	20.78	**<0.001**
	Hamstrings		49.41	18.69	55.49	7.46	**<0.001**
**Joint Power (W/kg)**	Ankle	Rt	2.15	0.29	3.01	0.54	**<0.001**
		Lt	2.21	0.36	2.93	0.43	**<0.001**
	Hip	Rt	1.21	0.44	1.89	0.501	**<0.001**
		Lt	1.21	0.33	1.98	0.11	**<0.001**
	Knee	Rt	1.28	0.51	1.97	0.24	**0.01**
		Lt	1.21	0.74	1.94	0.76	**0.01**
**Modified T test (s)**			14.66	3.44	12.69	1.6	**0.002**
**Proprioception (Score)**			42.92	11.86	36.97	9.35	< **0.001**
**Balance-single stance (mm)**	Left Foot	Antero-Posterior (A-P)	9.3	2.91	8.1	2.55	< **0.001**
		Medio-Lateral (M-L)	6.95	2.11	6.27	2.064	< **0.001**
	Right Foot	Antero-Posterior (A-P)	8.25	2.94	7.18	1.04	< **0.001**
		Medio-Lateral (M-L)	6.61	2.09	5.65	1.67	< **0.001**
**Reaction Time (s)**			2.18	0.19	1.7	0.11	**0.003**
**Hand-Eye Coordination (Score)**			46.53	11.17	58.4	7.13	< **0.001**
**Muscle Length Testing (Tightness)**			**Pre**				**Post**
	Iliotibial Band (ITB)		Mild				Normal
	Adductors		Mild				Normal
	Calf		Moderate				Mild
	Adductors		Mild				Normal
	Calf		Moderate				Mild
**Manual Muscle Testing (MMT)**	Biceps		4				5
	Triceps		4				4
	Rotator Cuff		4				5
	Quads		4				5
	Hams		4				5
	Calf		4				5
	Dorsiflexors		4				4

In the present study, we evaluated the lower limb peak muscle force for Quadriceps and Hamstring using the isokinetic device. The findings of the study showed that the mean peak force for the Quadriceps muscle increased from 66.18 Newton-meter (Nm) to 73.21 Nm in the WCN group which was statistically significant with a p-value of 0.001. Similarly, the Hamstring peak muscle force improved by 6.08 Nm. The increased peak force in the muscle could have been seen with the generation of higher force in an isotonic contraction recruiting a higher number of muscle fibers as a positive physiological response to the badminton sessions. Also, since the quality and velocity of movement could have improved at the lower limb joint following two weeks of regular badminton sessions, force generation could be better as a function of both mass and acceleration. These findings of the study agreed with previous findings. Although not reported in similar age groups, a study conducted by Yan and Li (2015) suggested improvement in speed among both men and women after badminton sessions. The findings of the study also suggested that the peak power at the hip, knee, and ankle improved significantly (p≤0.001) for WCN participants where the maximum change was seen at the ankle joint. The improved power at the lower limb could be attributed to both better force generation and increased joint velocity since power is a function of force and velocity. Findings from previous studies reported improvement in explosive strength and power following badminton training in both sex ( Fernandez *et al*. 2013, Lee *et al*. 2021, Mohammed 2020). The agility of the participants was evaluated using the Modified T test which is a reliable test for clinical assessment ( Sassi *et al*. 2009). In our study, we found that there was a mean reduction of 1.97 seconds in participants with non-communicable diseases. These findings suggest that the agility of the participants improved with the badminton sessions, and they conceded less time for effective movements with greater speed and quality. The improvement in agility could be attributed to the enhanced representational momentum (RM) as suggested by a previous study ( Jin *et al*. 2017). In addition, there were significant changes in proprioception, reaction time, balance, and hand-eye coordination. The proprioception was measured using the raw score and it improved significantly (p-value<0.001) with a reduction in mean score from 42.92 to 36.97. Single stance balance in millimeters (mm) was assessed for both limbs in anteroposterior and mediolateral directions and it was found that the balance improved significantly in both directions with a reduction in the mean covered area. These findings suggest that participants in the WCN group were able to balance themselves over shorter areas of contact more efficiently after the badminton session. The findings of the study were in line with previous study findings which suggested improved motor skills. The study conducted by Duncan *et al*. (2020) suggested improved quality and execution of motor skills following badminton sessions. Apart from the physical health benefits, studies have also reported that engagement in Badminton leads to enhanced mental health. In line with the previous studies, our study reported that the reaction time improved with a reduction in mean values which was also statistically significant (0.003) in addition to the hand-eye coordination score which improved significantly. These findings could be attributed to better concentration and the ability to choose the correct task and inhibit unnecessary movements. The study conducted by Takahashi and Grove (2019) compared the effects of badminton on inhibitory function comprising the ability to control attention or emotion to overcome a strong internal bias or external attraction and instead do what is most appropriate or necessary. Following the stop-signal paradigm developed by Logan (2014), our study suggested that participants could successfully inhibit their responses during the stop signal. Also, the study conducted by Hung *et al*. (2018) suggested consequent improvement in brain executive function as a result of higher levels of brain neurotrophic factor and better task-switching performance. For clinical findings, such as muscle length and strength, we found clinically significant changes in the WICN participants. The tightness of muscles such as the iliotibial band (IT band) and adductor reduced whereas the muscle strength improved (4 to 5) for both upper and lower limb muscles with manual muscle testing.

Table 3 represents findings for participants without non-communicable diseases (WICN). In contrast to WCN participants, WICN participants showed no significant changes in the shoulder range of motion. The findings were suggestive of lower mean changes in the pre- and post-session shoulder range of motion which is understandable in absence of any underlying condition that could have affected the joint range. However, like WCN participants, WICN participants showed a significant improvement in muscle peak force for both Quadriceps (Q) and Hamstring (H) (p value≤0.001 and 0.05, respectively). Considering the H/Q ratio for pre-session in WICN group, which was 0.67 and increased to 0.72 post-session, it can be suggested that the peak force improved significantly for the flexor and extensor group of muscles. The peak force was calculated for concentric quadriceps followed by concentric hamstring at a set torque of 60° sec-1 in the dominant leg. It has been suggested that a poor H/Q ratio is indicative of stronger quadriceps action compared to hamstring which could predispose the individual to knee injuries such as anterior cruciate ligament injury (ACL) due to increased anterior translatory force and poor coactivation of the hamstring to counter with stronger force ( Baratta *et al*.1988). The joint power for the ankle, hip, and knee improved significantly among the WICN participants with a maximum mean difference (approx. 1 W/kg) in the ankle joint. These findings were suggestive of better neuromuscular change and adaptation at the ankle joint which plays an important role in the dynamic stability of the human body. Similarly, findings for agility, proprioception, balance, reaction time, and hand-eye coordination showed significant differences among participants without the non-communicable disease (p-value≤0.05).

**Table 3.** Neuromuscular finding for participants without non-communicable disease (WICN).

Variables			Pre-Sessions		Post-Sessions		P value Sig (≤0.05)
			Mean	S.D.	Mean	S.D.	
**Shoulder Range of Motion (Degree)**	Flexion		179	2.8	180	0.64	0.14
	Extension		59.6	1.3	59.7	1.29	0.3
	Abduction		178	4.48	177	4.58	0.41
	ER		88	3.68	88	3.68	0.52
	IR		81.67	9.39	82.33	9.61	0.33
**Muscle Peak Force (Nm)**	Quadriceps		72.68	16.07	81.4	14.14	< **0.001**
	Hamstrings		49.4	10.25	58.7	9.83	**0.05**
**Joint Power (W/kg)**	Ankle	Rt	2.15	0.29	3	0.54	< **0.001**
		Lt	2.21	0.92	2.29	0.11	< **0.001**
	Hip	Rt	1.21	0.44	1.89	0.51	< **0.001**
		Lt	1.21	0.33	2.11	0.22	< **0.001**
	Knee	Rt	1.28	0.51	1.9	0.13	**0.01**
		Lt	1.21	0.74	2.3	0.78	**0.02**
**Modified T test (s)**			13.39	1.82	12.01	1.29	**0.001**
**Proprioception (score)**			39	6.97	34.54	5.95	< **0.001**
**Balance-single stance (mm)**	Left foot	A-P	9.06	1.52	8.05	1.45	< **0.001**
		M-L	6.91	2.37	6.19	1.93	**0.001**
	Right foot	A-P	8.59	1.86	7.64	0.88	**0.002**
		M-L	6.71	2.69	5.76	2.04	**0.003**
**Reaction Time (s)**			1.63	0.23	1.41	0.11	**0.005**
**Hand-Eye Coordination (score)**			60.33	9.58	70.47	8.91	**<0.001**
**Muscle Length**			**Pre**				**Post**
	ITB		Mild				Normal
	Adductors		Moderate				Mild
	Calf		Moderate				Normal
**Manual Muscle Testing (MMT)**	Biceps		4				5
	Triceps		4				5
	Rotator Cuff		4				4
	Quads		5				5
	Hams		5				5
	Calf		5				5
	Dorsiflexors		5				5

Table 4 compares the neuromuscular parameters between participants with and without non-communicable diseases. It was observed that there were statistically significant differences for a few parameters in the pre-session itself which become more after the post-session. For instance, the parameters that showed significant differences at the pre-badminton session included shoulder extension range of motion (p=0.003), Hamstring peak muscle force (p=0.03), proprioception (p=0.04), balance for the bilateral foot in the anteroposterior direction (p=0.02), and reaction time (p=0.01). In contrast to pre-session differences, post-session differences were observed in most of the variables including shoulder range of motion for extension (p=0.04), abduction (p<0.001), external rotation (p<0.001), and internal rotation (p=0.013), peak muscle for hamstring (p=0.025), joint power for the ankle (p<0.001) and knee (p=0.01), single stance balance in dominant limb (p=0.05), and reaction time (0.001).

**Table 4.** Comparison of Neuromuscular parameter changes post badminton session between participants with and without non- communicable disease (WCN Vs WICN).

Variables			Pre		Post	Effect size
			P value Sig. ≤0.05		P value Sig. ≤0.05	Cohen’s d (post session comparison)
**Shoulder Range of Motion (Degree)**	Flexion		0.152		0.62	0.06
	Extension		**0.003**		**0.04**	-0.32
	Abduction		0.603		**< 0.001**	0.71
	ER		0.356		**< 0.001**	0.49
	IR		0.302		**0.013**	0.64
**Muscle Peak Force (Nm)**	Quadriceps		0.317		0.251	-0.46
	Hamstrings		**0.03**		**0.025**	0.32
**Joint Power (W/kg)**	Ankle	Rt	0.09		**0.03**	0.37
		Lt	0.1		**< 0.001**	0.14
	Hip	Rt	0.25		0.06	0.17
		Lt	0.62		**0.03**	0.74
	Knee	Rt	0.43		**0.05**	0.36
		Lt	0.55		**0.01**	0.46
**Modified T test (s)**			0.14		0.36	0.46
**Proprioception (score)**			**0.04**		0.09	0.31
**Balance-single stance (mm)**		Left foot	A-P	**0.02**	0.07	0.02
			M-L	0.45	0.98	0.04
		Right foot	A-P	**0.02**	**0.05**	0.47
			M-L	0.31	0.53	0.06
**Reaction Time (s)**			**0.01**		**0.001**	1.08
**Hand-Eye Coordination (score)**			0.22		**0.03**	1.48

Considering the parameters that showed a difference in both pre- and post-badminton sessions such as shoulder extension range of motion, higher mean value changes were observed (58 to 59.7 degrees) for WICN participants in comparison to WICN participants where changes in mean values were subtle (59.6 to 60 degrees) suggesting that badminton session were more beneficial for participants with non-communicable disease towards improving shoulder range of motion. These findings were more evidently supported by the significant observed changes in the between-group analysis. The changes were significant between the NIC group to WCN at pre and post (p≤0.05), and not significant between NIC to WICN groups (p=0.218). Similar findings were observed for peak muscle force at hamstring, where the mean changes were higher for WCN participants compared to WICN participants (6.08 Nm and 3.28 Nm respectively). The peak quadriceps and hamstring muscle force for the NIC group at baseline was 71.99 Nm and 50.47 Nm respectively and there were insignificant changes at the post-session. Comparing the changes for WCN and WICN to the NIC group, both the WCN and WICN groups showed significant changes. Again, the WCN group showed a higher difference against the NIC group. These findings suggest that peak muscle force improved more in the participants with the non-communicable disease, nearly double compared to those without non-communicable disease considering the changes against the health control. The findings of this study also suggest that adaptation to imposed muscular strength demand among the WCN participants was more efficient with 8 weeks of badminton sessions as seen with a larger mean difference in peak force. However, it was noted that the peak force for Quadriceps between WCN and WICN groups didn’t show any significant difference for both pre- and post-session suggesting that power loss in hamstring could be more compared to quadriceps among older adults due to aging and underlying non-communicable disease. Since Quadriceps is used more over the Hamstrings for concentric contractions in daily activity, such findings may be observed which was also supported by the negative value of effect size (-0.4). Also, the H/Q ratio difference between the WCN and WICN groups pre- and post- session was not much suggesting that Quadriceps power remained proportionally equal to show any significant difference (H/Q ratio for WCN participants changed from 0.74 at pre-session to 0.75 at post-session whereas, for WICN participants, it changed to 0.67 to 0.68). The H/Q ratio for the NIC group at pre-session was 0.691 and 0.693 suggesting no changes in the pre-post value due to the absence of any vigorous physical activity. The *post-hoc* analysis suggested that there were no significant changes in the muscle power between NIC and WICN groups but significant changes between NIC and WCN groups as muscle power improved with the badminton session. It was interesting to observe that the proprioceptive changes were seen in the pre-session (p=0.04) between the WCN and WICN groups but not in the post-session (p=0.09) suggesting that proprioceptive feedback with badminton improved equally for both groups. The findings were supported by comparing both WCN and WICN groups to NIC which showed significant but similar changes in the *post-hoc* analysis. Due to the presence of non-communicable diseases such as diabetes, proprioception could have been affected much but the mean difference between the participants with and without non-communicable diseases at pre- and post-session (5.94 and 4.42) didn’t support such assumptions and with training, it improved equally and was not masked by the underlying disease. A significant difference was also observed for the single stance balance in antero-posterior direction at pre- and post-badminton sessions. Though mean changes were minimal but significant and it could be suggested that badminton sessions may help to improve the static and dynamic balance among older adults with and without non-communicable diseases as also suggested by previous studies which showed improvements in muscle coordination and balance among the elderly population ( Dube *et al*. 2015, Lam *et al*.2011).

The improvement in muscle coordination could also be supported by findings as seen with the reaction time which improved significantly between the groups. The mean difference change was seen better in WCN participants (2.18 seconds reduced to 1.7 seconds) compared to WICN participants (1.63 seconds reduced to 1.41 seconds). There was no significant reduction in the mean values for NIC groups (1.65 seconds). The *post hoc* analysis again suggested that more significant improvement was seen in the WCN group compared to NIC against WICN and NIC groups. The findings on balance, reaction time, and hand-eye coordination were clinically supported by the moderate effect size for balance in the dominant leg (0.47) and high effect size for reaction time and hand-eye coordination (1.08 and 1.48, respectively).

In addition, a few parameter parameters showed significant differences for post-session only. For example, the shoulder range of motion for abduction, and external and internal rotation showed a statistically significant difference between the groups where higher improvement was seen among WCN participants. These findings were not only statistically significant but also clinical differences could be suggested as supported by moderate to higher effect size for post-session changes (effect size - 0.49, 0.64, and 0.71 for shoulder external rotation, internal rotation, and abduction range of motion respectively). The joint power at the ankle, hip, and knee showed a statistically significant difference between the groups (p<0.001, p=0.03, and p=0.01 respectively). Improved joint power at post-session with a statistically significant difference could be suggestive of variations in the muscle synergy pattern during the dynamic motions. It is well known that ankle and hip synergy play a significant role in static and dynamic stability where ankle synergies are first to activate followed by the hip. Though joint power improved for both WCN and WICN groups at all three joints a significant post-session change is indicative of a larger difference in mean changes for the WCN group when compared to the NIC group. It can also be seen that the significant change in the hip joint power was supported by a moderate to high effect (0.74). In addition, the ankle and knee joints also showed improved power clinically with a moderate effect size of 0.34 and 0.46 respectively.

It is very evident from the above findings that important neuromuscular parameters not only showed a statistically significant difference between the groups but participants with the non- communicable disease showed better changes with higher changes in mean values.

Table 5 represents the comparison of cardiovascular parameters for within-group analysis. It was found that there were significant changes in the distance covered for the six-minute walk test following the badminton session. The mean change in 6MWD for the WCN participants was 129 meters for males and 118.06 for females (p≤0.001). It can be suggested that improvement through the difference in the distance covered for both males and females were similar although males covered more distance in pre-session compared to females for the non-communicable group ( Table 5). Improvement in the distance covered post-badminton session was suggestive of better cardiovascular function and endurance training over 8 weeks through Badminton. Literature reports an average 6 MWD of 400 to 700 meters for ages 40-85 years among healthy subjects. Our study reported lower mean values for both men and women at pre-session but it improved post-session and was seen within the suggested range (397 m to 526 m for males and 354 m to 472 m for females). The study conducted by Britto *et al*. (2013) suggested a reference value of 586± 106 m, 54 m greater in males compared to females whereas the study conducted by Al-Ghamdi and Shaheen (2021) among Saudi population aged 50-80 years suggested a reference value of 396.2±69.4 m. In our study, the mean value was in line with the study by Al Ghamdi and Shaheen (2021) but lower in comparison to Britto *et al*. (2013) for both pre- and post-session. We tried to compare our findings with the predicted values for 6MWD in previous literature using equations as given below:

$$6\;\mathrm{MWD}=1140\;m–\left(5.61\times \mathrm{BMI}\right)–\left(6.94\times \mathrm{age}\right)\;\text{for}\;\mathrm{men}$$

$$6\;\mathrm{MWD}=1017\;m–\left(6.24\times \mathrm{BMI}\right)–\left(5.83\times \mathrm{age}\right)\;\text{for women}$$

**Table 5.** Comparison of Cardiovascular parameters (within group analysis).

Groups	Variables	Pre		Post		P value
		Mean	S.D.	Mean	S.D.	
**Non-Communicable – Male (N=27)**	Six Minute walk distance (6MWD in meter)	397	54.80	526	38.12	<0.001
	Estimated VO _2_ peak **(ml kg** ^**-1**^ **min** ^**-1**^ **)**	14.07	3.28	17.03	4.01	<0.001
**Non-Communicable – Female (N=11)**	Six Minute walk distance (6MWD in meter)	354	49.32	472	45.49	0.002
	Estimated VO _2_ peak **(ml kg** ^**-1**^ **min** ^**-1**^ **)**	13.08	3.21	15.79	4.22	0.03
**Without Non-Communicable – Male (N=30)**	Six Minute walk distance (6MWD in meter)	425	49.32	567	52.03	<0.001
	Estimated VO _2_ peak **(ml kg** ^**-1**^ **min** ^**-1**^ **)**	14.71	4.37	17.98	4.99	<0.001
**Without Non-Communicable – Female (N=6)**	Six Minute walk distance (6MWD in meter)	369	45.54	496	43.17	0.01
	Estimated VO _2_ peak **(ml kg** ^**-1**^ **min** ^**-1**^ **)**	13.42	4.07	16.34	4.71	0.01

The calculation using the BMI in the above equation suggested a predictive 6MWD of 601 m for males and 574 m for females in our study. Comparing the above-given references suggested that people in UAE with the non-communicable disease could be at risk of cardiovascular events. Shorter 6 MWD has been correlated with poor cardiorespiratory function and direct predictor for cardiovascular events ( Tabata *et al*. 2014). If we consider the changes, the findings of our study suggest that badminton sessions not only improved cardiovascular function but could also be helpful to cease or reverse the course of non-communicable disease through improved physical activity and its direct correlation with enhanced cardiac and lung functions, thereby reducing the risk of non-communicable disease and its associated complications. The improvement in 6 MWD for WCN participants indicated a mean change of 142 m in males and 127 m in females. The mean difference for both males and females was higher in comparison to WICN participants which was also not only statistically significant between the WCN and WICN group but also in comparison to the NIC group (p < 0.001). Though the cardiovascular function in WCN improved most significantly, a higher mean for 6 MWD at pre- and post-session for the WICN group could be suggestive of better cardiovascular function in absence of non-communicable disease. Previous studies have reported 54-80 meters as a minimal clinical important difference (MCID) for 6MWD. Based on our findings, we can suggest that both WCN and WICN groups showed a difference in distance covered pre and post which is clinically significant reflecting improvement in cardiovascular function. Cardiovascular health is best determined using maximal aerobic capacity (VO _2_ max). Although the six-minute walk test cannot be considered the gold standard for VO2 max analysis, as a submaximal test good reliability has been reported for clinical and field testing ( Mänttäri *et al*. 2018). In our study, we used the six-minute walk test to estimate the VO _2_ max for both participants with and without non-communicable disease using a generalized equation (Mean Peak VO _2_ in ml.kg ^-1^.min ^-1^ = 4.948+0.023 x Mean 6 MWD in meters) which can be used to accurately estimate the mean peak VO _2_ ( Ross *et al*. 2010). Another study suggested estimation of VO2 max with demographic and 6 MWD with significant correlation as follows:

VO _2_ in ml.kg ^-1^.min ^-1^ = 12.701 + (0.06 × 6 MWD) - (0.732× BMI). Both equations gave similar results with a slightly higher value for the second equation. Since we did not find major changes in the pre- and post-BMI values, we choose the first equation for interpreting our results. The findings as given in Table 5, suggested that there was a significant improvement in the VO _2_ max for males and females in both WCN and WICN groups. In the WCN group, the mean value changed from 14.07 to 17.03 ml.kg ^-1^.min ^-1^ for males and 13.08 to 15.79 ml.kg ^-1^.min ^-1^ for females. Similar findings were seen for the WICN participants without the non-communicable disease. The pre- and post-changes in mean VO _2_ max values were statistically significant but less compared to changes in 6MWD as suggested by higher p values. The comparison between WCN and NIC was also more significant in comparison to the WICN and NIC group suggestive of better cardiovascular adaptation in the WCN group. For participants with and without non-communicable disease, the difference in mean peak VO2 was 3.04 ml.kg ^-1^.min ^-1^ and 2.7 ml.kg ^-1^.min ^-1^ respectively suggesting higher improvement among the non-communicable group. These findings were not only statistically significant but also showed clinical significance with reported MCID of 1.5–2.0 ml.kg ^-1^.min ^-1^ ( Blackwell *et al*. 2020). The improvement in peak VO _2_ following the badminton program could have been seen as a physiological adaptation of training over cardiovascular functions. Studies report that moderate to high-intensity exercise led to increased peak VO _2_ with increased oxygen update and metabolic equivalents ( Cabello *et al.* 2022). These findings are suggestive of improved cardiovascular endurance in response to exercise and training. Since the mean peak VO _2_ difference was greater in the WCN group compared to the WICN group, it can be said that more improvement can be expected in the non-communicable disease participants. Also, the higher difference in mean values between the groups could have been the attributing factor for the statistically significant difference in peak VO _2_ as shown in Table 6 (p=0.04 for males and 0.05 for females). Though the effect size was trivial for peak VO _2_ between the groups, 6MWD showed a higher effect size of 0.89 with a difference of 41 meters between the groups.

**Table 6.** Comparison of Cardiovascular parameters (between group analysis).

Groups	Variables	Difference in mean at pre-session	Difference in mean at post-session	P value	Effect size (post session comparison)
**Non- Communicable and without non- communicable (Male)**	Six Minute walk distance (6MWD in meter)	28	41	<0.001	0.89
	Estimated VO _2_ peak (ml kg ^-1^ min ^-1^)	0.64	0.95	0.04	0.21
**Non- Communicable and without non- communicable (Female)**	Six Minute walk distance (6MWD in meter)	15	24.19	0.01	0.55
	Estimated VO _2_ peak (ml kg ^-1^ min ^-1^)	0.34	0.55	0.05	0.31

## Conclusion

It was evident from the findings of this study that badminton sessions led to improved cardiovascular and neuromuscular function among older adults for both groups with and without the non-communicable disease. The mean changes were seen more in the non-communicable group which also showed statistical and clinically significant changes. Badminton can effectively engage older adults (males and females) to improvise their neuromotor skills and cardiovascular endurance, thereby could be useful as an alternative measure for physical activity in this age group across the globe. Policies can be made to inculcate sports driven physical interventional strategies to combat the non-communicable disease burden. The immediate impact can be seen in drawing the attention of the authorities and bring changes in the status of non-communicable diseases among the older adults of the UAE population with the introduction of such physical activity programs on a larger scale. The findings would encourage people to engage themselves in physical activity sessions and incorporate outdoor activities such as badminton in their daily routine for better body functioning.

## Data Availability

Figshare: BWF FULL RESEARCH DATA. https://doi.org/10.6084/m9.figshare.24448948.v1 ( Hazari, 2023). Data are available under the terms of the Creative Commons Attribution 4.0 International license (CC-BY 4.0).
