# Supplementary material for: Effect of 8 weeks badminton session on cardiovascular and neuromuscular functions among older adults in United Arab Emirates: a quasi-experimental study
Source: F1000Res. 2023 Nov 28;12:1522. [Version 1] doi: 10.12688/f1000research.142339.1 (PMC11184279; doi:10.12688/f1000research.142339.1)
Supplement: Peer review history

# Reviewer report: Reviewer response for version 1

**DOI:** 10.5256/f1000research.155881.r285267

**Published:** 2024-06-17

**Authors:** Zafar Azeem

**Affiliations:** 1 Dr. D.Y. Patil College of Physiotherapy,, Pimpri, Pune, India

The study looks promising considering the demands of badminton as a sport with targeting both aerobic and anaerobic requirements. However, the study lacks methodical rigor from a specific application or description of badminton training being administered to participants with NCDs. 

 The manuscript did not describe about any parameters for the badminton sessions, though the conclusion is drawn in support of such sessions to affect cardiovascular and neuromuscular function among older adults. 

 Participants with NCDs often face a challenge while participating in any demanding sports and physical activity. No such adverse events were reported. In addition, the discussion lacks scientific depth for support or against the study findings.

Is the work clearly and accurately presented and does it cite the current literature?

Yes

If applicable, is the statistical analysis and its interpretation appropriate?

Partly

Are all the source data underlying the results available to ensure full reproducibility?

No

Is the study design appropriate and is the work technically sound?

Yes

Are the conclusions drawn adequately supported by the results?

No

Are sufficient details of methods and analysis provided to allow replication by others?

Partly

Reviewer Expertise:

Sports Physiology, Exercise Science,Exercise Programming and Prescription, Sports Medicine and Physiotherapy, Sports biomechanics

I confirm that I have read this submission and believe that I have an appropriate level of expertise to confirm that it is of an acceptable scientific standard, however I have significant reservations, as outlined above.

# Reviewer report: Reviewer response for version 1

**DOI:** 10.5256/f1000research.155881.r226657

**Published:** 2024-05-11

**Authors:** Alia A. Alghwiri

**Affiliations:** 1 Department of Physical Therapy, School of Rehabilitation Sciences, The University of Jordan, Amman, Jordan

The study investigates the impact of an 8-week badminton intervention on cardiovascular and neuromuscular functions among older adults (40-70 years) in the United Arab Emirates, with a particular focus on those with and without non-communicable diseases (NCDs).

 The introduction underscores the vulnerability of older adults to cardiovascular and neuromuscular disorders due to insufficient physical activity, highlighting the potential of badminton as a means to enhance neuromuscular function. The study aims to analyze the effects of badminton on cardiovascular and neuromuscular function in older adults with and without NCDs.

 A total of 120 participants are recruited and divided into two interventional groups (with and without NCDs) and one non-interventional healthy control group. The badminton intervention involves 45-60 minute sessions thrice a week for two months, adhering to specific inclusion and exclusion criteria.

 The results of the study indicate a significant improvement in cardiovascular and neuromuscular variables both within and between the groups, with the greatest changes observed in participants with NCDs. The findings suggest that badminton can serve as an effective means to enhance cardiovascular and neuromuscular functions among older adults, with particular benefits for those with NCDs.

 The discussion and conclusion highlight the potential of sports like badminton in addressing the burden of non-communicable diseases among older adults. The immediate positive impact observed in participants with NCDs suggests the potential for such interventional sports activities to mitigate the burden of these diseases. The study emphasizes the importance of introducing such interventions on a larger scale to harness their potential benefits.

 Strengths of the study include its quasi-experimental design, specific focus on the older adult population, and the incorporation of participants with and without NCDs. However, limitations include the relatively short duration of the intervention and the absence of a follow-up to assess long-term effects.

 This study contributes valuable insights into the potential of badminton as an intervention for improving cardiovascular and neuromuscular functions among older adults, particularly those with NCDs.

 Future research could explore the sustainability of these improvements over a more extended period and investigate the mechanisms underlying the observed positive effects.

Is the work clearly and accurately presented and does it cite the current literature?

Yes

If applicable, is the statistical analysis and its interpretation appropriate?

Yes

Are all the source data underlying the results available to ensure full reproducibility?

No source data required

Is the study design appropriate and is the work technically sound?

Yes

Are the conclusions drawn adequately supported by the results?

Yes

Are sufficient details of methods and analysis provided to allow replication by others?

Yes

Reviewer Expertise:

Geriatrics

I confirm that I have read this submission and believe that I have an appropriate level of expertise to confirm that it is of an acceptable scientific standard.

# Reviewer report: Reviewer response for version 1

**DOI:** 10.5256/f1000research.155881.r226656

**Published:** 2023-12-15

**Authors:** Y. Ravi shankar Reddy

**Affiliations:** 1 Department of Medical Rehabilitation Sciences, College of Applied Medical Sciences, King Khalid University, Abha, Aseer Province, Saudi Arabia

The study is unique in terms of its sample population and application of sports among older adults. Most of the available researches focused in sports, address younger population. Having integration of sports as means of aerobic activity or physical activity could help to reduce the health burden of the nation.

 Introduction underlies and sets the background for the research question well.

 The methodology is well explained with valid and reliable outcome measures except few such as clinical examination for muscle length and strength testing.

 The results section address the important findings reflecting the tested hypothesis appropriately.

 Discussion of important findings are argued well in line with previous studies findings. New insights have been provided in between for justification of the findings which is impressive.

 Conclusion is concise and appropriate.

 Recommendations:

- List limitations of the study such as:
  1. Gender and age stratification which could have affected the results.
  2. Hyperglycemic and hypertension state and classification would could have affected their performance and the findings.
- Check for comprehension errors at certain places.
- Page no 13. Paragraph states comparison of WICN with WICN for shoulder extension range of motion which should be corrected.

Is the work clearly and accurately presented and does it cite the current literature?

Yes

If applicable, is the statistical analysis and its interpretation appropriate?

Yes

Are all the source data underlying the results available to ensure full reproducibility?

Yes

Is the study design appropriate and is the work technically sound?

Yes

Are the conclusions drawn adequately supported by the results?

Yes

Are sufficient details of methods and analysis provided to allow replication by others?

Yes

Reviewer Expertise:

Physical Therapy, Spine injury and rehabilitation, sports injuries and rehabilitation.

I confirm that I have read this submission and believe that I have an appropriate level of expertise to confirm that it is of an acceptable scientific standard, however I have significant reservations, as outlined above.
